# Test-Retest Reliability of Motor Function and Myometry Outcomes From the Vamorolone Trials in Duchenne Muscular Dystrophy

**DOI:** 10.1212/NXG.0000000000200289

**Published:** 2025-08-29

**Authors:** Rebecca A. Tobin, Eric P. Hoffman, Linda Johnson, Meredith K. James, Paula R. Clemens, Utkarsh J. Dang, the VBP15-LTE and VBP15-004 Investigators

**Affiliations:** 1 Carleton University, Ottawa, Canada;; 2 Department of Pharmaceutical Sciences, School of Pharmacy and Pharmaceutical Sciences, Binghamton University—State University of New York;; 3 TRiNDS LLC, Pittsburgh, PA;; 4 John Walton Muscular Dystrophy Research Centre, The Newcastle Upon Tyne Hospitals NHS Trust and Newcastle University, United Kingdom; and; 5 University of Pittsburgh School of Medicine and Department of Veterans Affairs Medical Center, Pittsburgh, PA.

## Abstract

**Background and Objectives:**

Understanding the reliability of outcomes used in clinical care and trials is important to delineate intervention-induced change from random variability. Reliability, sensitivity, and clinical meaningfulness of change are all key aspects of choosing the most appropriate outcome measure for a clinical trial. Common outcome measures to monitor progression and treatment effect in Duchenne muscular dystrophy (DMD) include measures of strength (myometry) and motor function tests: stand from supine velocity (STANDV), North Star Ambulatory Assessment (NSAA), 6-minute walk distance (6MWD), 10-m run/walk (RWV), and 4-stair climb velocity (CLIMBV). Our objective was to present test-retest reliability of common outcome measures using pretreatment measurements and to provide insights into missing measurements.

**Methods:**

Data were used on outcome measures in steroid-naïve, 4 to <7 years participants with DMD in 2 multisite, multicountry vamorolone trials (VBP15-002 [n = 48 participants] and VBP15-004 [n = 121 participants]). Bland-Altman analysis, the coefficient of variation, and the intraclass correlation coefficient (ICC) were used.

**Results:**

Based on the ICC, NSAA, RWV, and CLIMBV have good reliability, whereas the 6MWD and STANDV have moderate reliability. Two different techniques were used for myometry in VBP15-002 (CINRG Quantitative Measurement System [CQMS]) and VBP15-004 (MicroFET2 handheld digital muscle dynamometer [HHD]). Reliability of myometry ranged from poor to moderate, and CQMS did not show improved reliability over HHD. The 6MWD and myometry showed the highest rates of missingness. Because of similar age ranges and harmonized outcomes, our findings were compared with the FOR-DMD cohort. Myometry and CLIMBV were not in FOR-DMD; reliability findings were concordant for the 4 outcomes in common. For most outcomes, we found reduced reliability compared with previous studies in older age groups.

**Discussion:**

Reliable outcome measures, appropriate for the age of patients, are key to improving power in clinical trials, leading to fewer participants needed and reducing patient and site burden. We found NSAA, RWV, and CLIMBV to be the most reliable. This should be considered in the context of sensitivity to drug effect, and clinical meaningfulness of changes observed: all 5 motor outcomes were sensitive to drug effect of vamorolone and prednisone, whereas myometry was not.

## Introduction

Duchenne muscular dystrophy (DMD) is an X-linked genetic disorder affecting approximately 1 in 5,050 male births.^1^ DMD is caused by pathogenic variants of the *DMD* gene, leading to a lack of dystrophin, an essential protein for maintaining the cell membranes of muscle fibers.^2^ The lack of dystrophin causes ongoing myofiber degeneration and regeneration, progressive fibrotic replacement of skeletal muscle, loss of motor function and ambulation, and early death.

In clinical care and trials, disease progression in ambulatory patients with DMD is commonly measured via functional motor outcome measures, myometry, imaging (MRI), and patient-reported outcomes. The North Star Ambulatory Assessment (NSAA) is a Rasch-built motor function scale developed specifically for DMD.^3,4^ Other commonly used motor outcome measures include timed function tests such as the time to stand from supine (TTSTAND), time to run/walk 10 m (TTRW), and time to climb 4 stairs (TTCLIMB), as well as the distance walked in 6 minutes (6MWD).^5^ Myometry is commonly measured using isometric voluntary muscle contraction, for example, at the elbow (elbow extension [ELBEXT], elbow flexion [ELBFLX]), and knee (knee extension [KNEXT] and knee flexion [KNFLX]).

Understanding the reliability of these commonly used outcome measures is important to distinguish change over time accompanying disease progression or treatment effect from more random variability. Clinical trials of treatments can be complicated by small sample sizes (DMD is rare), the progressive nature of DMD, and variability of severity. The reliability of outcomes used in DMD has been previously reported.^6-11^ Methodologies for previous reliability publications typically fall within 2 settings: (1) a set of clinical evaluators each (once or several times) measured a group of participants with DMD to determine reliability and (2) pretreatment screening and baseline visits from a clinical trial allowed for a post hoc analysis of reliability.

Two clinical trials of vamorolone in DMD (ClinicalTrials.gov: NCT02760264, NCT03439670) provided independent cohorts with similar inclusion criteria, with harmonized outcome measures and assessment protocols that allow evaluation of reliability. VBP15-002 and VBP15-004 enrolled 48 and 121 steroid-naïve participants, respectively, aged 4 to <7 years. Screening and baseline visits from both studies allowed for evaluation of the reliability of study functional outcome measures.

Notably, functional motor outcomes and clinical evaluator training methods in the vamorolone trials were harmonized with those in the FOR-DMD trial (196 steroid-naïve participants aged 4 to <8 years; 32 sites in 5 countries).^12^ The reliability of motor outcome measures in the FOR-DMD trial was evaluated^11^ using pretreatment visits, allowing for direct comparison with the results reported herein; however, myometry outcomes were not available.

In this study, we present a more expansive reliability analysis than in previous early-age DMD studies by using a large sample from independent trials run in different years in multiple sites and countries (11 sites in 6 countries and 33 sites in 11 countries for VBP15-002 and -004, respectively). We also compare 2 systems for the measurement of myometry outcomes: the CINRG Quantitative Measurement System (CQMS; used in VBP15-002) and hand-held dynamometry (HHD; used in VBP15-004).

## Methods

### Participants

Data were from 2 trials, run by the Cooperative International Neuromuscular Research Group network: VBP15-002, an open-label, multiple-ascending-dose study of vamorolone (n = 48 enrolled) and VBP15-004, a randomized, double-blind, placebo-controlled and prednisone-controlled clinical trial of vamorolone (n = 121 randomized). Participants with genetically confirmed DMD in the trials were aged 4 to <7 years and steroid-naïve at screening. VBP15-002 required completion of the TTSTAND test without assistance; VBP15-004 required TTSTAND <10 seconds.

VBP15-002 had a 6-month extension (VBP15-003)^13^ followed by a 24-month extension (VBP15-LTE).^14^ Full descriptions of the study designs and ethics approvals of both trials were previously published.^13-17^

### Outcomes

Five motor outcome measures (TTSTAND, TTCLIMB, TTRW, 6MWD, and NSAA) were used in both VBP15-002/003/LTE and VBP15-004 (eAppendix1 for details on Training Plan of clinical evaluators). It has previously been shown that timed function tests in velocity fit distributional assumptions better as compared with seconds via residual Q-Q plots^18^ on top of better interpretation and use of imputed zero velocities for data missing because of disease progression. Hence, for TTSTAND, TTCLIMB, and TTRW, times were transformed to velocities for assessment of reliability (i.e., stand from supine velocity [STANDV], climb velocity [CLIMBV], and run/walk velocity [RWV], respectively). Isometric myometry tests conducted in both VBP15-002/003/LTE and VBP15-004 were ELBFLX and KNEXT while additional myometry tests conducted only in VBP15-002/003/LTE were ELBEXT and KNFLX. In VBP15-002/003/LTE, myometry was measured using CQMS.

The CQMS is a device specifically designed to measure maximum isometric force during muscle testing. It consists of a load cell integrated into a strapping system, which is anchored to a stable frame. This frame may be wall-mounted or attached directly to an examination table and provides a secure structure for participants to pull against during isometric contractions. Additional support and stabilization are standardized. Audio-visual feedback was used for motivation.

In VBP15-004, a MicroFET2 handheld digital muscle dynamometer (HHD) held by the evaluator was used to measure the isometric force exerted by the participant for the specific muscle to be tested.

Reliability analysis was done separately for these 2 methodologies; we label myometry that used CQMS with the suffix CQMS (e.g., KNEXT-CQMS) and myometry that used HHD with the suffix HHD (e.g., KNEXT-HHD).

### Data Analysis

Analyses were conducted in R including with the *ggplot2*^19^ and *irr*^20^ packages.

Screening and baseline visits provided 2 time points of outcome measure data over a short period (median difference of 16 and 28 days in VBP15-002 and VBP15-004, respectively). Outcomes that were only tested once pretreatment were removed. If a participant had 3 steroid-naïve visits with outcome measurements (screening, rescreening, and baseline), rescreening and baseline measurements were used; and if an outcome was measured at only 2 of the 3 visits, that pair was used.

Test-retest reliability analysis was conducted both with all data and with several clear outliers removed. Outliers were identified based on Bland-Altman plots, except for NSAA, where expert opinion of typical changes in NSAA scores within the pretreatment period was also used.

To assess repeatability, differences between the measurements at 2 time points were plotted against the mean of those values in Bland-Altman plots. The bias is given by the mean difference between repeated measures with a 95% CI.^21^ The 95% estimated limits of agreement describe the typical range of differences observed during repeat testing.^21^

The intraclass correlation coefficient (ICC) is a reliability index that quantifies the agreement and degree of correlation between repeated measures. The ICC provides a ratio of the between-subject variance over the total variance (between-subject and within-subject).^22^ The greater the proportion of the variation in outcome values that come from differences between participants, the higher the ICC. We used ICC(1,1) (one-way random effects, absolute agreement, single rater).^22,23^ Koo and Li^22^ advise using the following categories for ICC interpretation using the 95% CI lower bound: <0.5 is poor, 0.5–0.75 is moderate, 0.75–0.9 is good, and >0.9 is excellent reliability.

The coefficient of variation (%CV) provides the ratio of the within-participant SD over the mean. %CV has previously been reported in DMD studies.^9^ Herein, we used the root mean square method.^24^

### Reasons for Missingness

We also describe recorded explanations for (non–COVID-19 related) missing data in the context of long and taxing clinical trial visits and provide the number of instances in which certain outcomes that were scheduled to be measured were missing.

### Comparison With FOR-DMD

Because of harmonized protocols and similar age ranges (eTable 1), our findings were compared with reliability findings from the FOR-DMD cohort.^11^ Four outcomes were in common: NSAA, 6MWD, RWV, and STANDV. ICC values for all age groups combined were not provided for the FOR-DMD cohort; therefore, we compare ICC values across 3 common age groups: 4-<5 years, 5-<6 years, and 6-<7 years. The mean age between screening and baseline was used for reliability analysis. The ICC point estimates and lower bounds (upper bound not provided) from the FOR-DMD results were compared with the point estimates and CIs from the combined vamorolone cohort.

## Results

### Participants

Pretreatment screening and baseline visits from VBP15-002 (n = 48) and VBP15-004 (n = 121) were used for reliability analysis; all visits (including from VBP15-003 and LTE) were used for analysis of missing data. Data from the 2 latest, nonmissing, visits before day 1 of treatment were used and labeled as screening and baseline for simplicity. Only 4 of the 166 (2.4%) pairs of screening and baseline visits were affected by remote testing because of COVID-19, all because of remote baseline measurements of TTSTAND (eAppendix1 for more details).

The baseline characteristics of the 2 participant cohorts were similar (Table 1). The time difference between screening and baseline ranged from 2 to 69 days with a median of 24 days (interquartile range [IQR]: 19–28 days). The mean age between screening and baseline ranged from 4.03 to 7.04 years with a median of 5.34 (IQR: 4.71–6.18).

**Table 1:** Characteristics at Steroid-Naïve Baseline

Characteristic median (IQR)	Overall (n = 167)	VBP15-002 (n = 47)	VBP15-004 (n = 120)
Age^a^ (y)	5.34 (4.71–6.18)	5.11 (4.54–6.16)	5.41 (4.84–6.20)
Time between screening and baseline^b^ (d)	24 (19–28)	16 (11–21)	28 (22–30)
STANDV (rises/s)	0.20 (0.15–0.25)	0.21 (0.15–0.26)	0.20 (0.15–0.24)
CLIMBV (tasks/s)	0.23 (0.17–0.29)	0.23 (0.16–0.33)	0.22 (0.17–0.29)
RWV (m/second)	1.69 (1.42–1.96)	1.76 (1.49–1.92)	1.65 (1.40–1.96)
6MWD (m)	342.5 (298.8–373.5)	338.0 (291.0–382.5)	343.0 (301.0–370.0)
NSAA total score	19.0 (16.0–23.0)	19.5 (16.3–23.0)	18.5 (16.0–22.0)

Abbreviations: STANDV = stand velocity from time to stand test; CLIMBV = climb velocity from time to climb 4 steps test; IQR = interquartile range; RWV = run/walk velocity from time to run/walk 10 m test; 6MWD = six-minute walk distance; NSAA = Northstar Ambulatory Assessment.

a Mean age between screening and baseline.

b The time between screening and baseline was not always the same across all outcomes for a given participant (because of outcomes split between screening and rescreening or visits taking place over 2 d), so we instead pooled all nonmissing tests from all participants (n = 1,150; n = 368 from 002 and n = 782 from 004) and calculated the median from this larger distribution.

### Bland-Altman Analysis

**Figure 1:**
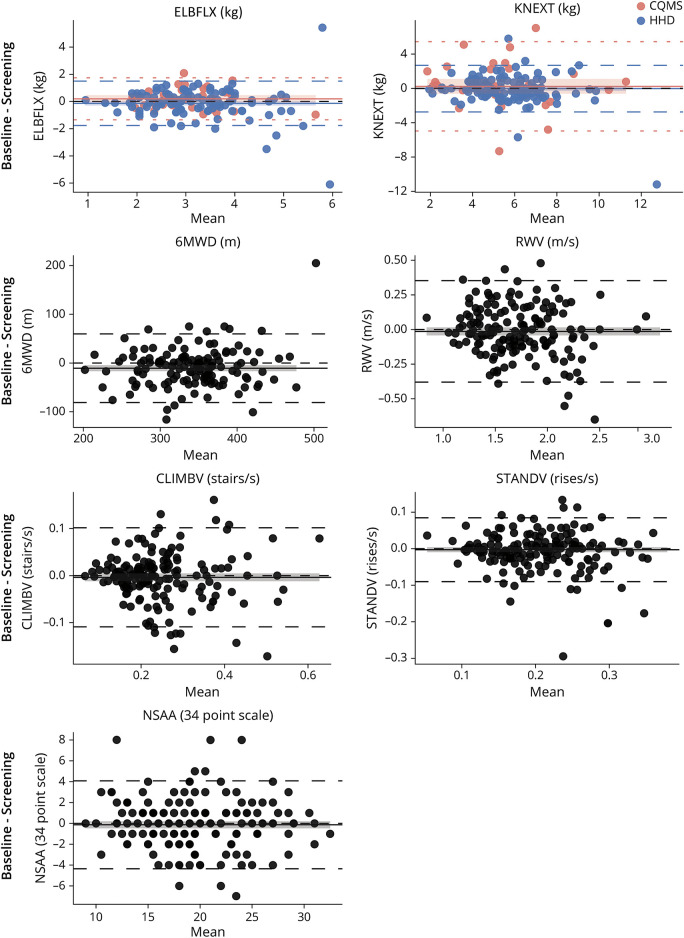
Bland-Altman Plots for Outcomes Present in Both the VBP15-002 and VBP15-004 Studies* Differences between baseline and screening are plotted against the mean between screening and baseline. Any bias is noted with a solid line; 95% CI for bias is given by a surrounding ribbon; 95% confidence limits of agreement given by dashed lines (outliers excluded in calculations). The bias and agreement limits for myometry outcomes were calculated separately for CQMS (orange) and HHD (blue); these are colored separately. Outliers are shown by boxes with a cross. *STANDV = stand velocity from time to stand test; CLIMBV = climb velocity from time to climb 4 steps test; RWV = run/walk velocity from time to run/walk 10 m test; 6MWD = six-minute walk distance; NSAA = Northstar Ambulatory Assessment; CQMS = CINRG Quantitative Measurement System; HHD = handheld digital muscle dynamometer.

Eleven outliers were identified via Bland-Altman analysis (Figure 1): one for 6MWD (Baseline—Screening = 205 m); 3 for STANDV (Baseline—Screening = −0.29, −0.20, −0.18 rises/s); 2 for ELBFLX-HHD (Baseline—Screening = 6.1, −5.4 kg); one for KNEXT-HHD (Baseline—Screening = −11.2 kg); 2 for KNFLX-CQMS (Baseline—Screening = −4.1, −3.5 kg); and 2 for ELBEXT-CQMS (Baseline—Screening = 2.7, 2.6 kg). In addition, 4 outliers were identified for NSAA (Baseline—Screening = 8, 8, 8, −7) based on expert opinion of typical changes in NSAA scores over a maximum 69-day pretreatment period.

The bias between screening and baseline was typically trivial as seen in the Bland-Altman plots (Figure 1). Bias was statistically significant although small for 6MWD: baseline distances were, on average, 10.6 m shorter (95% CI: −16.7 m, −4.5 m) than screening (possibly a false positive because of lack of multiple testing correction). For CLIMBV, larger variability was seen in repeated measures for participants who, on average, climbed faster.

### ICC

**Figure 2:**
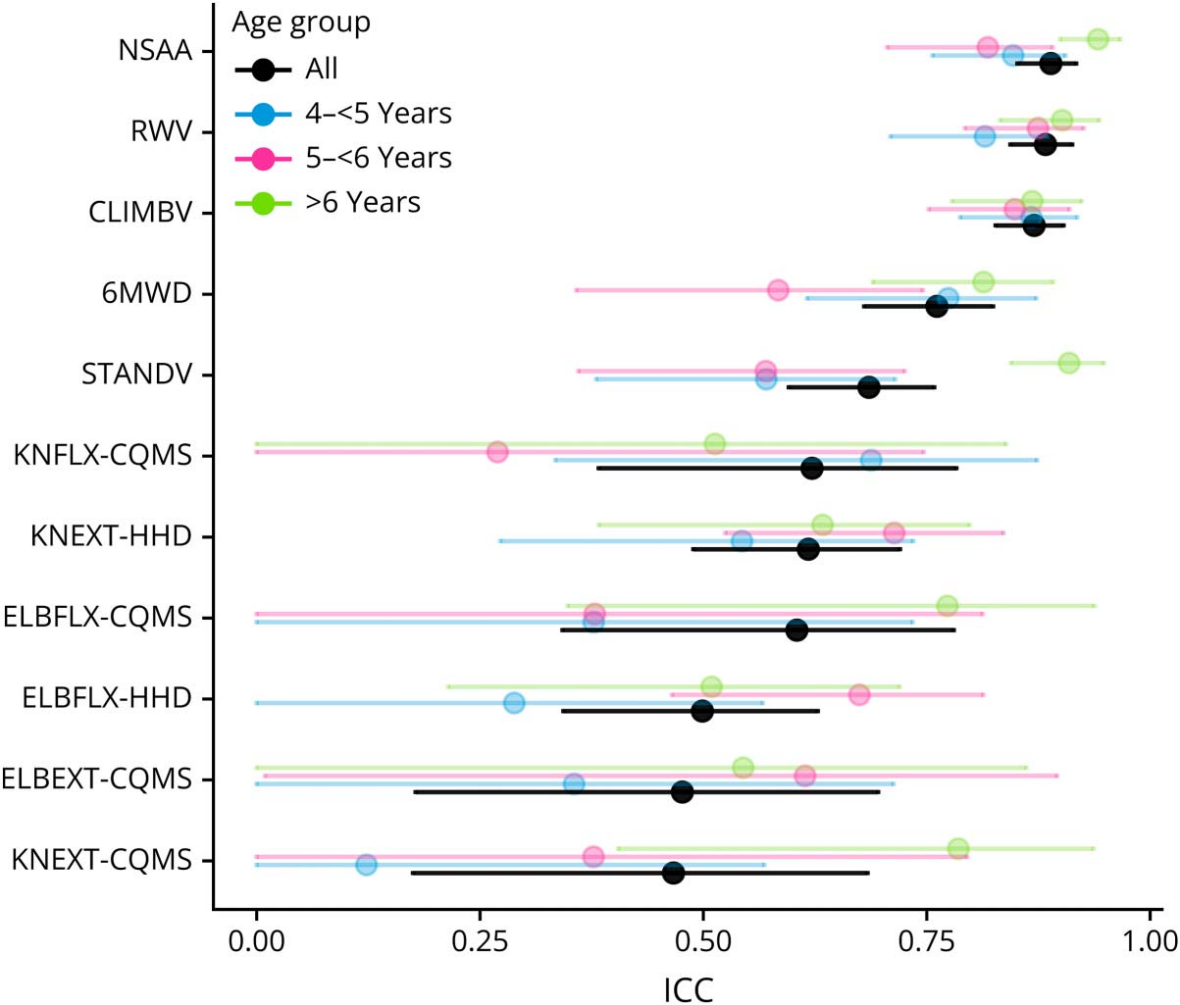
Intraclass Correlation Coefficient for Functional and Myometry Outcomes (Outliers Present)* Black: all participants; blue: 4-<5 year-age group; pink: 5-<6 year-age group; green: >6 year-age group. When the lower bound was calculated as negative, it was modified to be zero given a negative ICC is impossible. *STANDV = stand velocity from time to stand test; CLIMBV = climb velocity from time to climb 4 steps test; RWV = run/walk velocity from time to run/walk 10 m test; 6MWD = 6-minute walk distance; NSAA = Northstar Ambulatory Assessment.

In the following, we present analysis with outliers first, and then without outliers to show their impact on the ICC. Table 2 and Figure 2 show ICC values and 95% CIs. The reliability of outcomes was classified using the lower bound of the ICC. NSAA, RWV, and CLIMBV had good reliability; 6MWD and STANDV had moderate reliability; and KNFLX-CQMS, KNEXT-HHD, ELBFLX-HHD, ELBEXT-CQMS, ELBFLX-CQMS, and KNEXT-CQMS had poor reliability. Note that KNFLX-CQMS, KNEXT-HHD, and ELBFLX-HHD had moderate reliability with outliers removed (eFigure 1). For myometry outcomes present in both cohorts, higher ICC was obtained for HHD as compared with CQMS when outliers were removed; however, the CIs were wide and overlapping. Note that for myometry, CIs (and hence, the interpretations) relied on smaller sample sizes from a single trial given the 2 implementations used in different trials and the trial using CQMS had a much smaller sample size (Table 2).

**Table 2:** Reliability Measures With 95% CIs

Outcome	ICC (CI) (interpretation^a^) higher = better	%CV (CI) lower = better
NSAA total score (n = 162)	0.89 (0.85–0.92) (good)	9.6 (7.7–11.1)
−4 outliers	0.91 (0.88–0.94) (good)	8.3 (7.3–9.2)
RWV (n = 162)	0.88 (0.84–0.91) (good)	7.6 (6.7–8.4)
CLIMBV (n = 162)	0.87 (0.83–0.90) (good)	14.5 (12.6–16.2)
6MWD (n = 134)	0.76 (0.68–0.82) (moderate)	8.3 (6.9–9.6)
−1 outlier	0.79 (0.72–0.85) (moderate)	8.0 (6.7–9.1)
STANDV (n = 166)	0.69 (0.60–0.76) (moderate)	18.2 (14.5–21.3)
−3 outliers	0.77 (0.70–0.82) (moderate)	16.4 (13.8–18.6)
ELBFLX CQMS (n = 34)	0.60 (0.34–0.78) (poor)	17.7 (12.4–21.7)
ELBFLX HHD (n = 108)	0.50 (0.34–0.63) (poor)	20.4 (16.2–24.0)
−2 outliers	0.66 (0.54–0.76) (moderate)	18.3 (15.1–21.0)
KNEXT CQMS (n = 37)	0.47 (0.17–0.68) (poor)	37.1 (23.1–47.2)
KNEXT HHD (n = 112)	0.62 (0.49–0.72) (poor)	18.3 (14.0–21.8)
−1 outlier	0.70 (0.59–0.78) (moderate)	17.5 (13.3–20.9)
ELBEXT CQMS (n = 35)	0.48 (0.18–0.70) (poor)	22.9 (17.0–27.6)
−2 outliers	0.61 (0.34–0.78) (poor)	20.5 (15.0–24.9)
KNFLX CQMS (n = 38)	0.62 (0.38–0.78) (poor)	27.8 (21.5–32.9)
−2 outliers	0.72 (0.52–0.85) (moderate)	26.3 (19.7–31.5)

Abbreviations: STANDV = stand velocity from time to stand test; CLIMBV = climb velocity from time to climb 4 steps test; RWV = run/walk velocity from time to run/walk 10 m test; 6MWD = six-minute walk distance; NSAA = Northstar Ambulatory Assessment; ELBEXT = elbow extension; ELBFLX = elbow flexion; KNEXT = knee extension; KNFLX = knee flexion; CQMS = CINRG Quantitative Measurement System; HHD = handheld digital muscle dynamometer.

Where applicable, %CV and ICC values were calculated with and without outliers.

a ICC interpretations from Koo and Li (2016).

Data from younger age groups showed worse reliability, with the clearest differences between age groups occurring for the STANDV test in our cohort. On the other hand, the ICC for CLIMBV was seemingly the least affected by age (Figure 2, eTable 2). Note that for TTCLIMBV, residuals were less concordant with the assumptions of the ICC model compared with other outcomes. Bootstrapping of the ICC led to a slightly wider CI for TTCLIMBV ([0.82–0.91]), indicating that assumption deviations were not consequential. Findings on reliability as a function of duration and same vs different clinical evaluator are provided in eAppendix1.

### %CV

Table 2 also shows %CV estimates and 95% CIs. The impact of outliers and time between repeated measures were similar to ICC, but the trend of improved reliability with age was no longer consistent (a clear pattern was evident for STANDV, but CIs were overlapping; eTable 3).

### Proportions of Missing Values and Causes of Missingness Across Outcomes

To better understand participant response and refusal as well as the usability of outcomes including training and equipment aspects, all longitudinal data on outcomes for all dosed participants were used. Proportions of (non–COVID-19 related) missingness were evaluated and noted explanations of missingness (and counts thereof) described in Table 3 and eTable 4. STANDV, RWV, CLIMBV, and NSAA had few missing values (∼0.7–1.2% of total possible measurements), with the least missing being CLIMBV. The 6MWD was the most missing outcome, with 118 missing values (11.9% of possible measurements).

**Table 3:** Explanations of Missingness Across Outcomes (Non–COVID-19 Related), Categorized Into Themes^a^

	NSAA	RWV	CLIMBV	6MWD	STANDV	ELBFLX	KNEXT
Unclear	2	1	1	7	6	50	50
Attention/behavior issue	2	2	0	49	2	9	4
Inability to follow or understand directions	1	0	0	51	0	9	6
CE/physio not available or not certified	3	3	3	3	5	4	4
Equipment/software issue	0	0	1	0	0	7	5
Refusal	0	0	0	0	0	5	2
Injury	0	1	1	1	1	3	0
Disease progression	2	0	0	4	0	0	0
Other^b^	0	1	1	3	1	1	0
Total missing (not because of COVID-19)	10	8	7	118	15	88	71
Percent missing of total possible measurements (not impacted by COVID-19), (%)	1.0	0.8	0.7	11.9	1.2	8.8	7.1

Abbreviations: STANDV = stand velocity from time to stand test; CLIMBV = climb velocity from time to climb 4 steps test; RWV = run/walk velocity from time to run/walk 10 m test; 6MWD = 6-minute walk distance; NSAA = Northstar Ambulatory Assessment.

a The themes were curated after reconciling information from multiple sources.

b Reasons for missing with 2 or fewer occurrences listed in eTable 1.

Myometry outcomes, ELBFLX, and KNEXT, had a 13-fold and 10-fold increase in the proportion of missing outcomes compared with TTCLIMBV, with 88 and 71 missing values (8.8% and 7.1% of total possible measurements), respectively. This increase in missingness was largely driven by CQMS data: 16.2% and 13.6% of ELBFLX and KNEXT measurements were missing for CQMS compared with 4.3% and 3.2% for HHD, respectively.

Table 3 shows the explanations of missingness categorized into themes; 117 missing outcomes were missing with no reason provided, and this was predominantly an issue with myometry testing. “Attention/behavior issue” and “inability to follow or understand directions” were the next most common reasons, leading to 68 and 67 missing values, respectively. These reasons were most common for the 6MWD but were also overrepresented in myometry testing.

The proportion missing decreased with older age at baseline—8.0% (227/2,842) for 4-<5 years, 4.2% (106/2,521) for 5-<6 years, and 3.6% (96/2,662) for >6 years. The 2 causes of missingness related to participant behavior, attention, and comprehension, occurred relatively less frequently with older age, with 64.4%, 24.4%, and 11.1% of those occurrences happening in 4-<5 years, 5-<6 years, and >6 years, respectively.

### Comparison With FOR-DMD Study

Because of harmonized protocols, our findings were compared with findings from the FOR-DMD cohort (n = 196).^11^ Inclusion criteria included the ability to complete the TTSTAND test independently. One outlier was removed for RWV. Table 4 shows ICC values, separated by age group, for the FOR-DMD and VBP15 cohorts. FOR-DMD did not include the TTCLIMB test and myometry, leaving 4 outcomes for comparison. Note that a single ICC estimate across the entire range for each outcome was not provided^11^; ICC values were provided separated by age groups. ICC values were generally concordant between the VBP15 cohorts and the FOR-DMD cohort.

**Table 4:** Comparison to Analysis of FOR-DMD Trial Data Report^11^

		VBP15 trials			FOR-DMD trial		
Outcome	Age group	ICC	CI	n	ICC	Lower bound^b^	n
NSAA	4-<5 y	0.85	0.76–0.91	60	0.76	0.61	36
	−1 outlier	0.88	0.80–0.92	59			
	5-<6 y	0.82	0.71–0.89	53	0.88	0.82	62
	−3 outliers	0.88	0.80–0.93	50			
	6-<7 y^a^	0.94	0.90–0.97	49	0.86	0.77	38
	7–8.1 y	N/A	N/A	N/A	0.92	0.87	27
RWV	4-<5 y	0.82	0.71–0.89	60	0.68	0.50	35
	5-<6 y	0.87	0.79–0.93	53	0.86	0.79	60
	6-<7 y^a^	0.90	0.83–0.94	49	0.68	0.51	37
	7–8.1 y	N/A	N/A	N/A	0.84	0.72	27
6MWD	4-<5 y	0.77	0.62–0.87	41	0.53	0.28	31
	5-<6 y	0.58	0.36–0.75	46			
	−1 outlier	0.66	0.46–0.80	45	0.77	0.67	58
	6-<7 y^a^	0.81	0.69–0.89	47	0.73	0.57	36
	7–8.1 y	N/A	N/A	N/A	0.84	0.72	27
STANDV	4-<5 y	0.57	0.38–0.71	64	0.67	0.48	35
	−1 outlier	0.63	0.45–0.76	63			
	5-<6 y	0.57	0.36–0.73	54	0.72	0.60	59
	−2 outliers	0.73	0.57–0.84	52			
	6-<7 y^a^	0.91	0.84–0.95	48	0.76	0.62	37
	7–8.1 y	N/A	N/A	N/A	0.92	0.85	27

Abbreviations = STANDV = stand velocity from time to stand test; CLIMBV = climb velocity from time to climb 4 steps test; ICC= intraclass correlation coefficient; RWV = run/walk velocity from time to run/walk 10 m test; 6MWD = six-minute walk distance; NSAA = Northstar Ambulatory Assessment.

a For the VBP15 trials, there were 3 patients who had their seventh birthday between screening and baseline. Here, these 3 participants were included in the 6-<7-year group. Note that in the previously published paper^11^, 1 outlier was removed for the FOR-DMD data, and ICC values were only provided with this outlier removed.

b Upper bound not provided.

## Discussion

VBP15-002 and VBP15-004, clinical trials of vamorolone, provided 2 independent, young cohorts (4-<7 years of age at screening) with repeated steroid-naïve visits to assess the reliability of commonly used motor and strength outcomes in DMD.

Reliability of motor functional tests ranged from moderate to good, with NSAA, RWV, and CLIMBV having good reliability, and 6MWD and STANDV having moderate reliability based on the ICC. More generally, selecting outcomes with higher ICCs values in trials will lead to improved statistical power,^25,26^ and a reduction in sample size requirements, in the minimal clinically important difference, because of lower within-patient measurement variability, and an increase in the signal-noise ratio.

Four outcomes were in common with a recent publication on the FOR-DMD cohort^11^: NSAA, 6MWD, RWV, and STANDV. Based on the proximity of FOR-DMD ICC point estimates within our 95% CIs, only small differences were seen—most notable 6MWD in the 4-<5 years group and RWV in the 6-<7 years group, where reliability in FOR-DMD was lower—however, CIs were wide and overlapping. Across all age groups, NSAA was the most reliable outcome in both FOR-DMD and VBP15-004. ICC values for all age groups combined were not provided by the published report^11^ Also, note that small sample sizes because of separating by age group led to wider CIs. Samples sizes were larger for the VBP15 cohort for the 4-<5-year and 6-<7-year age groups. We also confirmed the finding that the time between repeated measures did not impact the ICC.

Note that for both the FOR-DMD and vamorolone trials, the most severe patients were excluded at screening; these studies required that participants be able to complete the TTSTAND test, with VBP15-004 additionally requiring that TTSTAND not exceed 10 seconds. Hence, our findings are not applicable to more severely affected individuals with DMD. Relevant to this is the finding that participants with 3′ gene variants affecting all dystrophin isoforms may be under-represented in clinical trials because of disease severity and cognitive involvement.^18^

The 6MW and TTSTAND tests are more physically demanding compared with other tests. Our results suggest that in this age range, reliability worsens with test difficulty, likely because difficult tests are more affected by day-to-day fluctuations in individual motivation and fatigue. Although TTSTAND is one of the tests in the NSAA, it is scored on a 3-point scale (based on modification of task vs not) and is 1 of 17 subtests, which likely reduces the impact of day-to-day variability on the NSAA total score.

For myometry outcomes, reliability was assessed separately for the 002 and 004 cohorts given the methodological differences (i.e., CQMS vs HHD). Reliability for all myometry outcomes was poor (poor to moderate) based on the ICC with outliers included (excluded). Previous studies of the reliability of myometry in boys with DMD had found more favorable ICC values, although the age range of participants extended into the teens.^6,7^ It is also notable that the Food and Drug Administration (FDA) recommends myometry as an appropriate and reliable outcome to be used in clinical trials of children 5 years and older.^27^ Studies of myometry reliability in adults with other neuromuscular conditions (myotonic dystrophy type 1 [n = 19], spinal muscular atrophy [n = 33], and inflammatory myopathy [n = 50]) have found ICC values ranging from moderate to excellent,^28-30^ as categorized by a reference article.^22^

Previously, a study of 30 boys (ages not provided) with DMD evaluated by a combination of 9 experienced clinical evaluators found the reliability between HHD and CQMS to be comparable.^31^ However, in the current study, myometry reliability from HHD was better than CQMS (for both ELBFLX and KNEXT), although CIs were wide and overlapping. This suggests that in these multicountry, multisite trials, CQMS was not superior in reliability compared with hand-held myometry.

CQMS uses a computer-based game as visual motivation to achieve maximal participant effort, whereas HHD only uses verbal coaching; however, it requires lengthy set-up both before and between each muscle group, and this waiting time may affect attention and motivation in young children. CQMS requires specialized software, training, and wall-mounted equipment, leading to greater expense.^32^ The increased time taken to perform CQMS also can limit its suitability in clinical trials. Note as well that twice as many muscle groups were tested in the study that used CQMS, which may have added to fatigue.

For STANDV, older age was associated with higher ICC. ICC tended to increase with increasing age across most outcomes; however, the CIs in these subgroups overlapped. Boredom, difficulty following rules, decreased attention span, and fatigue during a long visit can also affect performance. It is also often hypothesized that older children, more familiar with such tests in the clinic, may have less of a learning effect because of repeated testing. It is interesting to note that we saw a statistically significant bias between baseline and screening measurements only for 6MWD in the Bland-Altman analysis; however, this bias was clinically small (10.6 m longer at screening, on average). This indicates that this learning effect did not lead to systematically improved performance in our cohort.

The %CV values showed similar relative patterns among outcomes to the ICC.

In the VBP15 trials, myometry outcomes did not show efficacy of vamorolone in a short time period (e.g., 6 months), unlike the functional outcome measures, which did.^14^ Visual analysis of longitudinal trajectories revealed that functional motor outcome measures more consistently differentiated between high-dose vs low-dose treatment vs placebo compared with myometry. It is possible that myometry outcomes are not affected by efficacious treatments. It is also possible that myometry outcomes would only be significant in a clinical trial setting with a large enough sample of participants or a different time horizon; however, because of large random fluctuations between measurements, myometry outcomes were not significant in the VBP15 trials. This latter possibility is more likely given that strength-based outcomes, although other implementations/methodologies, have been significant in other studies of glucocorticoids.^33^ Given these findings, we recommend against using myometry (via a CQMS or HHD implementation) in clinical trials in this age group unless a larger acute treatment response is expected compared with steroids.

There were little (non–COVID-19 for VBP15-004) related missing data in these trials overall. However, the 6MWD, ELBFLX, and KNEXT tests had much more missing data than other outcomes. Outcomes were 1.2 times and 2.2 times more likely to be missing in the 5-<6-year and 4-<5-year age groups compared with the >6-year age group. The 6 MW and myometry tests were conducted following the other 4 tests done in the sequence of function/strength tests. The 6MWD was most often missing because of attention and comprehension reasons suggesting this test may be more difficult and/or complex than other tests for younger boys. These reasons were also more common for myometry than the other functional tests, although quite often (non–COVID-19 related), the cause was unclear for these outcomes. It is notable that although the 6MWD and STANDV had similar ICC values and are both considered difficult tests, the STANDV had a much lower rate of missingness. This suggests that the length of the test is another key factor.

A strength of this study is that reliability is assessed within the context of clinical trials and therefore reflects typical time differences between repeated measures, realistic differences between evaluator training across different sites and countries, and the measurement of outcomes during long and emotionally challenging visits to the clinic. In studies of reliability outside of a clinical trial setting in which sets of clinical evaluators repeatedly measure a group of participants to determine reliability (i.e.,^6,7,10^), the ICC is typically found to be excellent.

A previous study^8^ used screening and baseline (not strictly steroid-naïve) measurements from an international multicenter randomized, placebo-controlled trial of ataluren in 174 ambulatory boys age≥5 years, to present ICC (but no CIs) for 6MWD, RWV, CLIMBV, and STANDV. Their ICC values were higher for all outcomes except RWV, with 6MWD and STANDV having the largest differences. That study used hand-held myometry and measured both the left and right sides rather than only the dominant side (as was done in the VBP15 trials). Comparing with VBP15-004, which also used hand-held myometry, outcomes in common were ELBFLX and KNEXT but ICC values for both the right and left sides were higher in the previous report. Differences in the eligibility criteria used in this previous report may explain the improved reliability found in that study, especially the larger age range of 5–20 years. Given our finding that reliability tends to increase with higher age, one would expect improved reliability in an older age group. This is also supported by missing data reasons relating to participant's behavior, attention, and ability to understand instructions being more frequent in younger ages.

Although we show NSAA, RWV, and CLIMBV to be the most reliable motor outcomes, it is important to consider other key factors in choosing a primary outcome for a drug registration trial—namely, sensitivity for drug effect, clinical meaningfulness, natural history of the enrolled cohort, and study duration. For sensitivity to drug effect, an outcome can be highly reliable, but insensitive to drug effect, thus making it a poor motor outcome to use in a clinical trial setting.

Factors that contribute to insensitivity include ceiling effect, where it is difficult or impossible to detect an improvement if the outcome score is at the top of the range of values. Also, in older patients with DMD, muscle tissue is lost, and if the target tissue of the drug is muscle (typically true), the outcome may be insensitive to drug effect because of loss of target tissue. In this context, it is important to point out that all 5 motor outcomes studied were highly sensitive to both vamorolone (6 mg/kg/d) and prednisone (0.75 mg/kg/d) in the 24-week, double-blind, placebo-controlled trial (VBP15-004).^15^ For the lower dose of vamorolone tested in the same trial (2 mg/kg/d), 4 of the 5 outcomes tested met statistical significance for drug effect, with only RWV failing to meet statistical significance—despite this outcome being the second most reliable as shown here. Thus, RWV can be said to be highly reliable, but less sensitive to drug effect than other motor outcomes. It is also useful to note a newer, validated digital end point that captures free living activity rather than peak performance at a clinical site by measuring the 95th percentile stride velocity^34^ and has shown good ICC values in 5–7 years of age (ICC: 0.956) although compliance in wearing the device was significantly limited at some time points.^35^ Also of note, none of the strength outcomes (myometry) were sensitive to drug effect in any of the vamorolone trials.

There is also the consideration of clinical meaningfulness. All motor outcomes in DMD can be considered surrogate outcomes for how a patient feels, functions, or survives. Significant improvements in this with respect to a drug-related change can be a reasonably compelling argument for clinical meaningfulness. Whether there is regulatory precedence for acceptance of changes in a motor outcome as clinically meaningful is also important to consider. For the 5 motor outcomes in DMD studied here, the FDA and European Medicines Agency have deemed vamorolone-related changes in TTSTAND velocity and givinostat-related changes in TTCLIMB as clinically meaningful and hence warranting drug approvals. Thus, although reliability may differ (data shown here), these have the most regulatory precedence in terms of regulatory acceptance of clinical meaningfulness. Going forward, Sponsors may consider building a rationale for clinical meaningfulness of 10 m run/walk, given that it is likely the most reliable motor outcome. However, building such an argument may be challenging because changes in the 10 m run/walk, effectively a “sprint”, may be less clearly connected to how a patient functions as compared with, for example, TTSTAND.

A wide range of reliability was found for commonly used outcomes in a young age range in DMD. NSAA, RWV, and CLIMBV all had good reliability and low proportions of missing data. The 6MWD had moderate reliability and a higher rate of missingness, especially at younger ages—with issues with attention, behavior, and understanding test instructions being the most common causes. STANDV had moderate reliability, with worse reliability in the 4-<5-year age group compared with older age groups. The smaller differences in ICC values between age groups and similar results in a previous report^8^ (for RWV and CLIMBV), which occurred in a larger age range, both suggest the reliability of these outcomes is less affected by age. Myometry outcomes ranged from poor to moderate reliability and had a higher rate of missing data, especially at younger ages. The CQMS did not have improved reliability over hand-held myometry.

Given the FDA's recent guidance that trials of disease-modifying drugs should be performed in younger age groups,^27^ the findings of this study are of particular importance to ensure that age-appropriate outcomes are used. These findings are important in clinical trials and care and may help in reducing patient and site burden.

## Glossary

CLIMBV: climb velocity
CQMS: CINRG Quantitative Measurement System
%CV: coefficient of variation
DMD: Duchenne muscular dystrophy
ELBEXT: elbow extension
ELBFLX: elbow flexion
FDA: Food and Drug Administration
HHD: handheld digital muscle dynamometer
ICC: intraclass correlation coefficient
KNEXT: knee extension
KNFLX: knee flexion
NSAA: North Star Ambulatory Assessment
STANDV: stand from supine velocity
TTCLIMB: time to climb 4 stairs
TTRW: time to run/walk 10 m
TTSTAND: time to stand from supine
